# A Rare Case of Euglycemic Diabetic Ketoacidosis: Triad of Influenza A, Methicillin-Susceptible Staphylococcus aureus Pneumonia, and Sodium-Glucose Co-transporter 2 Inhibitor Use

**DOI:** 10.7759/cureus.90882

**Published:** 2025-08-24

**Authors:** Ofek Raviv, Nimra Tejeda, Issa Ali, Osama Khan, Jared Piotrkowski

**Affiliations:** 1 Internal Medicine, Cleveland Clinic, Weston, USA; 2 Clinical Sciences, Ross University School of Medicine, Bridgetown, BRB

**Keywords:** euglycemic dka, influenza virus type a, insulin drip, methicillin-sensitive staph aureus, methicillin-sensitive staphylococcus aureus (mssa), pneumonia, sglt-2 inhibitor, superimposed bacterial

## Abstract

Euglycemic diabetic ketoacidosis (euDKA) is a rare but life-threatening complication of diabetes characterized by ketoacidosis with only mildly elevated or normal blood glucose levels. Sodium-glucose co-transporter 2 (SGLT2) inhibitors are recognized precipitants, particularly during acute illness. We report, to our knowledge, a previously unreported triad: euDKA precipitated by influenza A with secondary methicillin-susceptible *Staphylococcus aureus* pneumonia in a 66-year-old man with type 2 diabetes on long-term SGLT2 inhibitor therapy. Despite near-normal glycemia on presentation and a history of previously tolerated mild respiratory infections on this regimen, he was found to have high anion gap metabolic acidosis (pH: 7.27) with markedly elevated serum ketones. He was managed with intravenous insulin, dextrose, fluids, and concurrent treatment of influenza and bacterial pneumonia, resulting in full recovery. This case underscores that life-threatening ketoacidosis can develop despite near-normal glucose and prior tolerance of acute illness and highlights the importance of early recognition, standardized treatment, and “sick day” precautions for patients taking SGLT2 inhibitors.

## Introduction

Diabetic ketoacidosis (DKA) is an acute, life-threatening complication of diabetes mellitus (DM) characterized by hyperglycemia, ketosis, and metabolic acidosis. Euglycemic DKA (euDKA) denotes ketoacidosis with near-normal or only modestly elevated glucose and accounts for an estimated 2.6% to 3.2% of DKA presentations [1]. Reports of euDKA have increased in parallel with the use of sodium-glucose co-transporter 2 (SGLT2) inhibitors [2]. SGLT2 inhibition lowers plasma glucose via glycosuria but can favor ketogenesis through relative insulinopenia, glucagon excess, and volume depletion [1,2]. As SGLT2 inhibitors entered the market, the US Food and Drug Administration issued a safety communication in 2015 regarding DKA with SGLT2 inhibitors [3]. Subsequent pharmacoepidemiology studies have shown a two to threefold higher DKA risk after SGLT2 initiation than with other agents [4]. Currently, a substantial proportion of SGLT2-associated DKA cases present with glucose <250 mg/dL, which can delay recognition and treatment [2,5].

Infection, ischemia, reduced carbohydrate intake, surgery, pregnancy, and other stressors commonly precipitate euDKA [1,2]. Influenza can further predispose to severe secondary bacterial pneumonia, particularly with *Staphylococcus aureus* [6]. We report a previously unreported triad in this context: euDKA precipitated by influenza A with secondary methicillin-susceptible *S. aureus* (MSSA) pneumonia in a patient on long-term SGLT2 inhibitor therapy. This case highlights a diagnostic vulnerability created by near-euglycemia and underscores practical “sick day” management for patients using SGLT2 inhibitors.

## Case presentation

A 66-year-old man with a history of type 2 DM (glycated hemoglobin: 9.7%), diagnosed five years before, and untreated hypertension presented in mid-May to the ED with a four-day history of fever, productive cough with yellow sputum, pleuritic chest pain, and progressive dyspnea. He also reported generalized weakness, nausea, and poor oral intake for two days. His home medications included metformin 1000 mg twice daily, empagliflozin 10 mg daily, and lisinopril 20 mg daily. He had been taking metformin for the past five years and empagliflozin for the past two years. He had no history of DKA. He weighed 87.1 kg (BMI: 27.49), never smoked tobacco, and reported social alcohol use (<14 drinks/week, <4 drinks/day). He had not received the influenza vaccine for the 2024-2025 flu season. He reported prior upper respiratory infections while on the same medications, but none required hospitalization.

**Figure 1:**
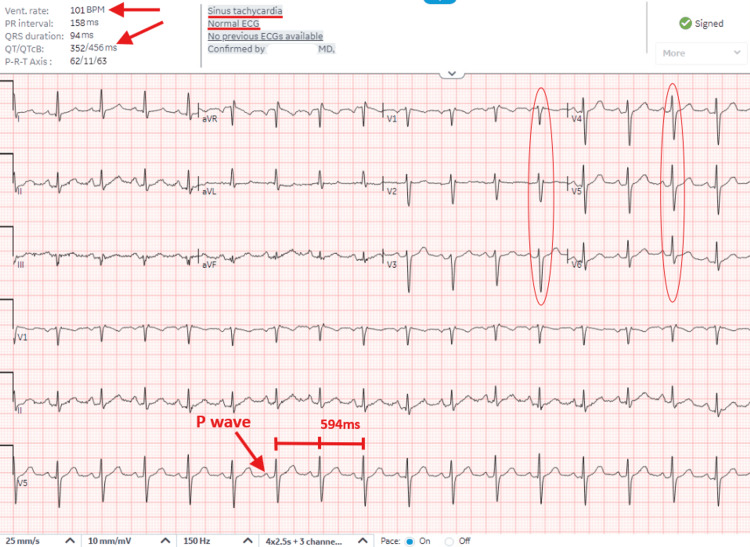
EKG taken on the day of admission showed normal sinus tachycardia with a mildly elongated qTC for an adult male at 456 ms. Highlighting points include a heart rate of 101, the presence of a P-wave before all QRS complexes, and a regular rhythm with equal distance between QRS complexes in lead V5 at the bottom of the EKG. Circled in red ovals are areas of good R wave progression, with exemplary QRS complexes increasingly rising above the isoelectric line from V1 (septal lead) to V6 (lateral lead). This was a grossly normal EKG. This interpretation was confirmed by the reading EP physician on call. EKG: Electrocardiogram; EP: Electrophysiology; P-wave: Represents atrial depolarization; QRS complex: Represents ventricular depolarization; qTC: Corrected QT interval; V1: Ventricular chest lead number 1; V5: Ventricular chest lead number 5; V6: Ventricular chest lead number 6

On initial evaluation, the patient appeared ill and mildly confused. His vital signs revealed a temperature of 37.9°C (100.2°F), a blood pressure of 160/72 mmHg, a heart rate of 102 beats/min, a respiratory rate of 26 breaths/min with deep, sighing respirations, and an oxygen saturation of 92% on room air (improving to 97% on two liters of nasal cannula). Physical examination was notable for dry oral mucous membranes, Kussmaul breathing, and right basal lung crackles. Cardiac examination showed tachycardia without murmurs. Abdominal examination was soft with mild diffuse tenderness. Bedside capillary glucose was 151 mg/dL. An electrocardiogram on admission (Figure 1) showed sinus tachycardia at 100-110 bpm, with non-specific ST-T wave changes but without an acute ischemic pattern. Initial laboratory studies demonstrated a serum glucose of 229 mg/dL, a venous pH of 7.27, and a beta-hydroxybutyrate of 6.01 mmol/L (reference<0.4). Serum electrolytes revealed sodium of 132 mEq/L, potassium of 4.7 mEq/L, and chloride of 95 mEq/L; blood urea nitrogen was 21 mg/dL, creatinine was 0.76 mg/dL, and serum lactate was 1.3 mmol/L. Urinalysis showed 4+ ketones and 4+ glucose. Hemoglobin was 15.1 g/dL, and leukocyte count was 19.86×10³/µL (85% neutrophils), and polymerase chain reaction testing of a nasopharyngeal swab was positive for influenza A. High-sensitivity troponin T was normal.

**Figure 2:**
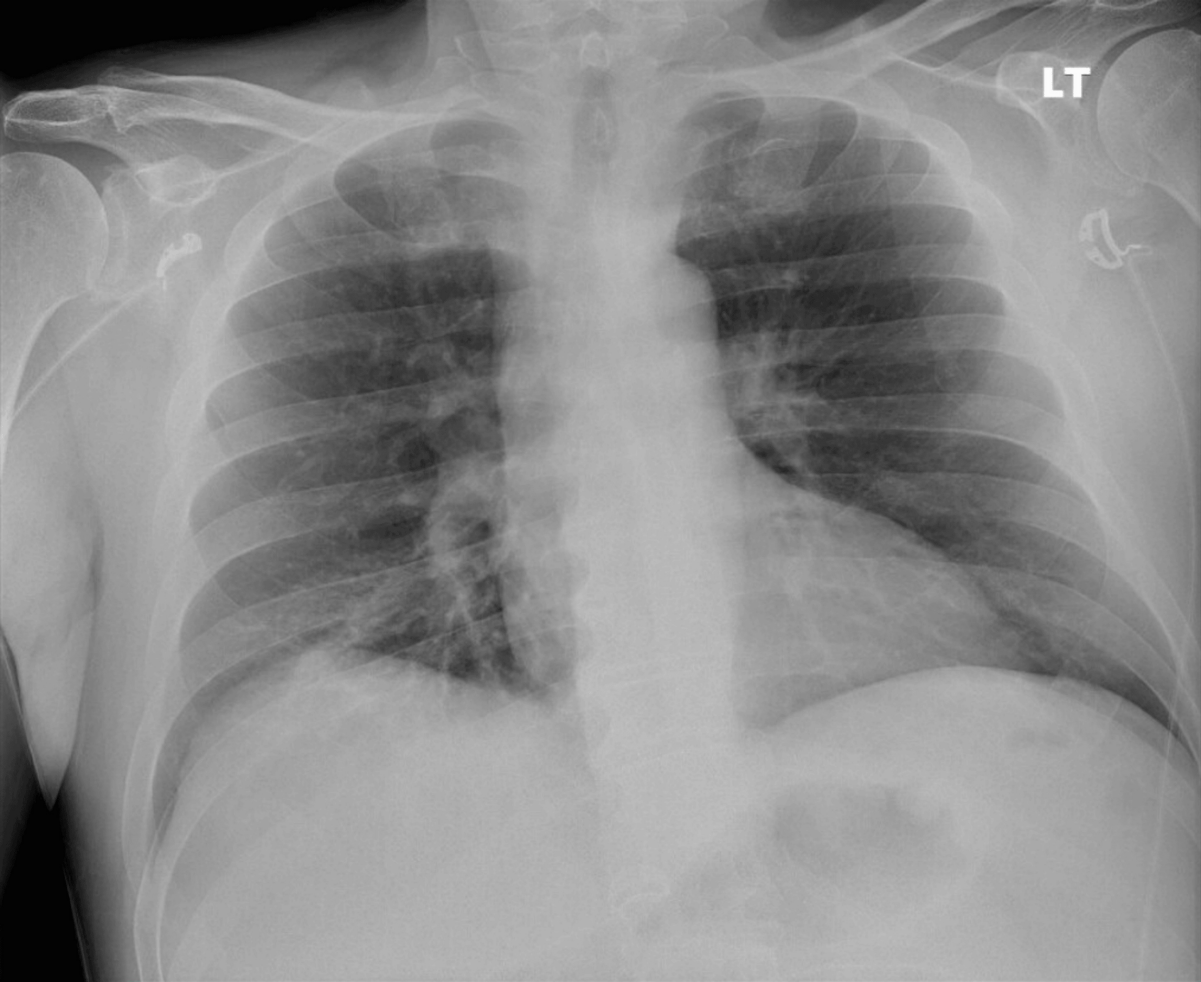
CXR taken on the day of admission showed no significant intrathoracic abnormality, with normal bilateral lungs and a normal-sized heart. This was confirmed by the radiological read and reviewed by the primary team physician. CXR: Chest X-ray

**Figure 3:**
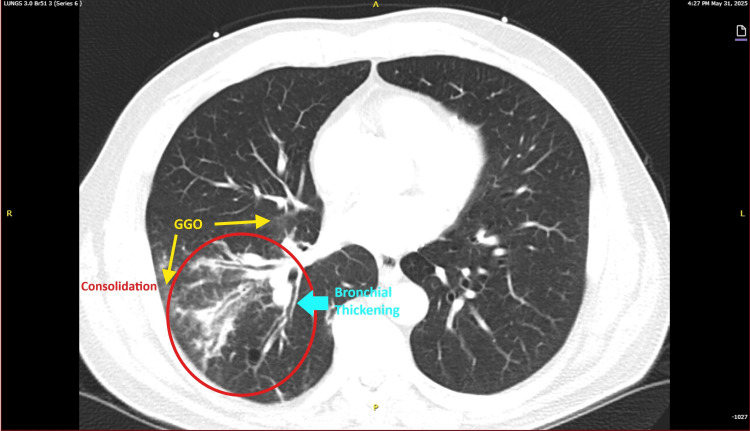
CTA chest, axial view (Series 6, Image 123) obtained on the day of admission shows a right lower lobe consolidation circled in red, consistent with superimposed bacterial MSSA pneumonia. GGO within and surrounding the consolidation are highlighted in yellow and likely represent viral influenza A pneumonia. Bronchial wall thickening related to the combined inflammatory processes is indicated in teal. Findings were confirmed by radiological interpretation. CTA: Computed tomography angiography; GGO: Ground glass opacities; MSSA: Methicillin‑sensitive *Staphylococcus aureus*

**Figure 4:**
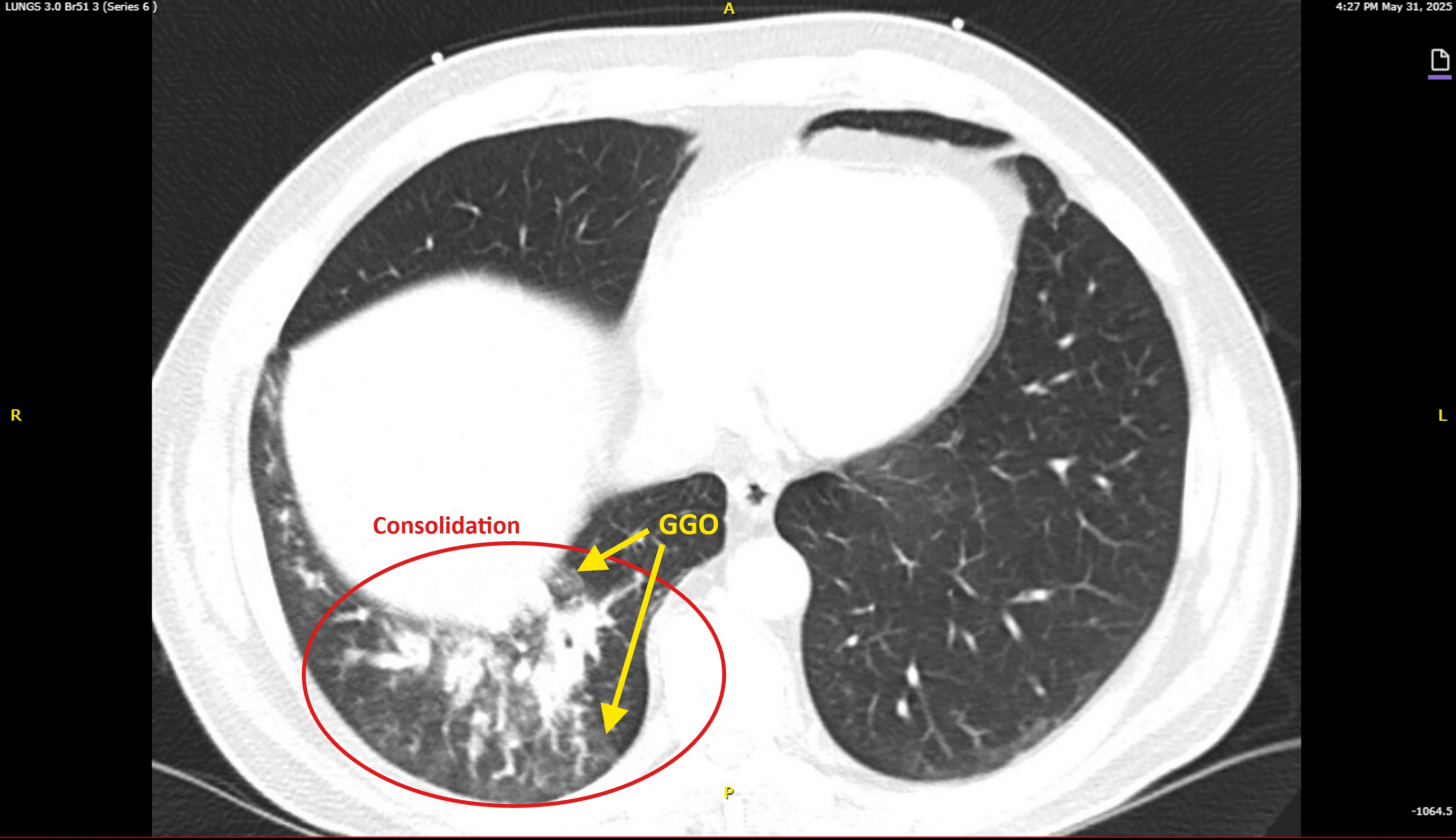
CTA chest, axial view (Series 6, Image 148) taken at a lower thoracic level than Figure 3 demonstrates dense posterior right lower lobe consolidation circled in red, representing the extension of lobar bacterial MSSA pneumonia. Associated GGO highlighted in yellow are consistent with viral influenza A pneumonia. Findings were confirmed by radiological interpretation. CTA: Computed tomography angiography; GGO: Ground glass opacities; MSSA: Methicillin‑sensitive *Staphylococcus aureus*

A chest radiograph (Figure 2) demonstrated no acute intrathoracic abnormality. Follow-up imaging was obtained in the ED to further evaluate the extent of pneumonia and to rule out complications such as abscess or empyema, given the severity of infection and persistent fever. A contrasted chest computed tomography (CT) showed consolidation of the right lower lobe with no significant pleural effusion or abscess. Representative axial CT slices of the lung fields (Figures 3, 4) demonstrate dense consolidation in the right lower lobe with air bronchograms, consistent with lobar pneumonia. No cavitation was noted within the consolidation.

Given the patient’s influenza infection and now present superimposed bacterial pneumonia, he was started on vancomycin and ceftriaxone along with oseltamivir. Despite the relatively normal blood glucose, the presence of high anion gap metabolic acidosis of 25 with elevated ketones and acidemia led to a diagnosis of euDKA, likely precipitated by the viral infection and compounded by reduced oral intake and ongoing SGLT2 inhibitor therapy. The patient was admitted to the ICU, and the SGLT2 inhibitors were discontinued. An intravenous (IV) insulin drip was initiated alongside aggressive IV fluid resuscitation. Because of the patient’s near-euglycemia, dextrose 5% in half-normal saline was co-administered to prevent hypoglycemia while continuing insulin infusion to clear ketones. Electrolytes were closely managed, with IV potassium supplementation given as the serum potassium was below 5 mEq/L and would be shifted intracellularly with the onset of insulin therapy.

Over the first 12 hours in the ICU, the patient’s acidosis began to correct (anion gap closed by ~36 hours after therapy initiation). His mentation and hemodynamics improved with treatment. On hospital day two, antibiotics were de-escalated further to IV ceftriaxone alone, once the nasal methicillin-resistant *S. aureus* (MRSA) swab resulted in the detection of only MSSA. At that time, other cultures were still pending; ceftriaxone was continued, given concern for other community-acquired pathogens such as *Streptococcus pneumoniae*. A sputum culture, obtained on the day of admission, grew moderate gram-positive cocci in clusters, and identified as *S. aureus* with susceptibility to oxacillin and cefoxitin. Blood cultures were negative throughout admission. By the end of the second hospital day, the patient's respiratory support requirement decreased from four liters to two liters of oxygen. He received respiratory therapies (nebulized bronchodilators and chest physiotherapy) at least every eight hours of each day.

By hospital day three, the patient’s anion gap metabolic acidosis had fully resolved, and he was transitioned from IV insulin to subcutaneous insulin glargine, with a sliding scale regimen. His blood glucose remained in the 140-180 mg/dL range on subcutaneous insulin, and ketosis did not recur. The influenza A infection was managed supportively with oseltamivir for five days. The superimposed lobar bacterial pneumonia showed marked clinical improvement; the patient defervesced by day four and was weaned off supplemental oxygen. The patient was transferred to the general medical ward and discharged with oral antibiotics, an injectable insulin regimen, and without SGLT2 inhibitors on his medication list. He was advised to avoid SGLT2 inhibitors in the future, given his DKA episode, and to promptly seek medical care for any signs of metabolic decompensation.

## Discussion

EuDKA in this patient was defined by high anion gap acidosis with ketosis and glucose of 151-229 mg/dL, a pattern that can obscure timely diagnosis [1,2,5]. Across DKA cohorts, evaluation should also consider myocardial ischemia when clinically relevant, particularly in older adults with poorly controlled type 2 diabetes and hypertension, as in our patient [7,8].

What distinguishes this case is the convergence of long-term SGLT2 inhibitor therapy, influenza A, and secondary MSSA pneumonia. This combination aligns with the known propensity of influenza to predispose to severe bacterial superinfection with *S. aureus*. It explains the patient’s initial need for broad-spectrum coverage before de-escalation to ceftriaxone once MRSA screening revealed MSSA, along with a congruent sputum culture [2,6].

The pathophysiology underlying SGLT2 inhibitor-associated euDKA likely played a central role in this case. Glycosuria induced by SGLT2 inhibition reduces plasma glucose but also promotes a relative reduction in serum insulin, an increase in glucagon secretion, and impairment of renal ketone clearance. When compounded by reduced caloric intake and the catabolic stress of dual infection, these mechanisms manifested a perfect storm for systemic ketoacidosis [1,2]. This is supported by β-hydroxybutyrate of 6.01 mmol/L with an anion gap of 25 and a normal lactate of 1.3 mmol/L.

Practical implications illustrated in this case include prompt ketone and anion gap assessment when symptoms suggest DKA despite modest glucose, early dextrose administration to allow continued insulin until ketonemia and the gap resolve, and explicit sick day guidance to hold SGLT2 inhibitors during acute illness or reduced intake [1,3,9].

## Conclusions

To our knowledge, this case presents a previously unreported triad: euDKA precipitated by influenza A with secondary MSSA pneumonia in a patient on multi-year SGLT2 inhibitor therapy. This highlights that life-threatening ketoacidosis can develop despite near-normal serum glucose and prior tolerance of intercurrent illnesses. Key lessons include early consideration of euDKA in atypical presentations, prompt measurement of ketones and anion gap, initiation of standard DKA therapy with early dextrose to permit continued insulin, and systematic identification and treatment of precipitating causes, particularly viral illness with possible secondary bacterial pneumonia. Equally important are practical preventive strategies: clear “sick day” guidance to hold SGLT2 inhibitors during acute illness or reduced intake, patient education on warning symptoms, careful discharge planning with a basal-bolus insulin regimen when indicated, and lifelong avoidance of SGLT2 inhibitors after a DKA episode. Recognizing this unique constellation and acting decisively can shorten time to treatment, prevent complications, and improve outcomes; these skills should extend beyond endocrinology to all frontline clinicians who care for acutely ill adults with diabetes.
